# A randomised controlled pilot trial of two interventions to manage dry mouth in pre-operative elective surgical patients

**DOI:** 10.1186/s40814-020-00630-0

**Published:** 2020-06-24

**Authors:** Leesa Morton, Amanda Tsan Yue Siu, Samuel Fowler, Chen Zhou, Christopher Nixon, Doug Campbell

**Affiliations:** 1 Department of Anaesthesia, Canterbury District Health Board, 2 Riccarton Avenue, Christchurch Central, Christchurch, 8011 New Zealand; 2 Department of Anaesthesia, Counties Manukau District Health Board, 100 Hospital Road, Otahuhu, Auckland 2025 New Zealand; 3 Department of Anaesthesia and Perioperative Medicine, Auckland District Health Board, Level 8, Support Building, Auckland City Hospital, Park Road, Grafton, Auckland 1023 New Zealand

**Keywords:** Dry mouth, Xerostomia, Anaesthesia, Artificial saliva, Pilot, Feasibility, Randomised, Pre-operative, Treatment, Patient centred outcome, Elective surgery

## Abstract

**Background:**

Dry mouth is a common perioperative patient complaint. There are a number of treatments used for dry mouth in other settings which are effective. None have been tested previously in the perioperative setting. Interventions to Manage Dry mouth (IM DRY) compared the effect of water and a saliva substitute on mouth dryness. The primary objective was to demonstrate the feasibility of conducting a large randomised controlled trial and secondary scientific aims were to assess treatment potential efficacy.

**Methods:**

Single blind, pilot randomised controlled trial (RCT) of 101 pre-operative elective surgical patients who were randomised to water or saliva substitute (Biotene oral rinse, GlaxoSmithKline, Australia) at a tertiary, university hospital. Dry mouth was assessed by 100 mm visual analogue scale (VAS) and 5-point Likert score.

**Results:**

One hundred participants completed follow-up and comprised the analysis dataset. All feasibility outcomes were achieved (recruitment rate > 5 participants a week, >95% completeness of the dataset, study protocol acceptability to staff, acceptability to participants > 66% and adherence to time limits within the protocol). Mean recruitment rate was 6 participants per week. These data were 99% complete. There were no adverse side effects or complications noted. There were no concerns raised by staff regarding acceptability. Overall, there was a mean of 30 min (± SD 5 min) between delivery of the intervention and the assessment, 30 min being the target time. The difference in VAS post intervention was − 11.2 mm (95% CI − 17.3 to − 5.1 mm) for water and − 12.7 mm (95% CI − 18.7 to − 6.7 mm) for saliva substitute. The proportion of patients who had improved dry mouth increased from 52% for water to 62% for saliva substitute.

**Conclusions:**

IM DRY successfully achieved its primary feasibility aims: recruitment rate, completeness of these, acceptability and protocol adherence. Saliva substitutes, used in the perioperative management of dry mouth, may be a simple, inexpensive, and low risk solution to help alleviate this common complaint. A large randomised controlled trial is feasible and is currently recruiting (ANZCTR 12619000132145).

**Ethics and Trial registration:**

Northern A New Zealand Health and Disability Ethics Committee (reference 17/NTA/152). Australian New Zealand Clinical Trials Registry (Number: 12618001270202). Registered retrospectively 18 October 2018.

## Background

Dry mouth is a symptom frequently reported pre-operatively [1]. It may be associated with thirst due to fasting and associated dehydration, use of pharmacological agents, stress and anxiety [2]. In the pre-operative setting, dry mouth and thirst can be difficult to separate. A recent study has shown thirst to be the most common complaint in post-operative patients. The SNAP-1 study in the UK identified thirst as the most prevalent type of severe discomfort post operatively (18.5%) [1]. Patient reported outcome measures such as pain, anxiety and nausea have multiple therapeutic options. Dry mouth is an important, but often overlooked symptom, with few proven therapeutic options.

Dry mouth, or xerostomia, is amenable to a number of therapies such as saliva substitutes, topical saliva promoters and intravenous sialogues [3–5]. There is some evidence for these treatments for symptom control in the chronic dry mouth associated with radiotherapy, autoimmune conditions (for example, Sjogren’s syndrome) and the use of some medications (for example, anticholinergics) [3]. None of these treatments have previously been tested in a perioperative setting.

Saliva substitutes are topical agents which provide a moisture retaining coat over the oral mucosa reducing the unpleasant sensation of dry mouth. They are the simplest and cheapest therapeutic option in this context. There is some evidence that they reduce symptoms in the context of chronic xerostomia [6]. There are a number of preparations available over the counter in the form of lozenges, sprays, mouth rinses, gels, oils, chewing gums and pastes. Dry mouth and thirst are prevalent in both the pre-operative and postoperative periods. We have chosen to investigate potential efficacy in preoperative patients, as symptoms will not be confounded by intraoperative fluid losses and replacement, antisialogue medications and pain.

The primary aim of the IM DRY pilot study was to assess the feasibility of conducting a larger randomised control trial (RCT) comparing two interventions to treat dry mouth in the pre-operative period. The primary feasibility outcomes were assessment of recruitment rate, acceptability of the intervention to participants, data completeness and adherence to the protocol. Secondary scientific outcomes were to assess the potential efficacy of two interventions to reduce the sensation of dry mouth in pre-operative patients.

## Methods

We conducted a prospective, single-blind randomised controlled pilot trial of two interventions to manage dry mouth in pre-operative patients. Randomisation ratio was 1:1. Written informed consent was sought prior to participation.

### Feasibility outcomes

Feasibility outcomes included assessment of recruitment rate, acceptability of the intervention to participants and staff, completeness of these and adherence to the protocol. Our decision to proceed to a larger trial was based on achieving all five of the feasibility outcomes.

1. Recruitment rate: Aim for greater than 5 Participants recruited per week.
2. Acceptability of the intervention to participants: Assessed by recording reasons for declining to participate. Participants were also asked if they would have the intervention again, and free text feedback was requested. A greater than 66% participant agreement to have the intervention again was seen as meeting this criteria.
3. Acceptability of the intervention to staff: Assessed by requesting feedback from the staff involved.
4. Completeness of data: The completeness of these data was deemed adequate if 95% of all trial endpoints were complete.
5. Adherence to the protocol: Assessed by keeping a recruitment log and reviewing each participant for protocol violations. Also, assessed by evaluating if time between intervention and assessments were at study target of 30 min.

### Secondary outcomes

The potential efficacy of two interventions to reduce dry mouth in pre-operative patients was secondary scientific outcomes in the IM DRY pilot trial. Assessment was by a 100-mm visual analogue scale (VAS) with the anchor words ‘not dry at all’ at 0 mm and ‘worst dry mouth imaginable’ at 100 mm. There are no formally validated scales or scoring systems specifically designed to assess dry mouth that were appropriate in this perioperative setting. However, a number of studies in this area have utilised modified VAS [7, 8]. Three options for the assessment scale were reviewed by fifteen pre-operative patients to assess comprehension and usability/ease of use in this setting before deciding on the VAS used in the pilot trial. Similar scales have been used in intensive care patients [9] and in patients with xerostomia [10]. VAS was delivered immediately before the intervention. Thirty minutes after administration of the intervention, patients completed a post-intervention questionnaire. This included the same VAS as used pre-intervention and in addition, participants were asked on a Likert scale, ‘How did your dry mouth feel after the treatment?’ (worse, no change, better or much better), and comparison between groups was made between the proportions of participants whose dry mouth improved (better or much better). The difference between the pre-intervention VAS and post-intervention VAS was calculated.

### Study setting and participants

Recruitment took place in the day of surgery admissions unit at Auckland City Hospital over a period of 16 weeks between October 2017 and February 2018. Participants were required to comply with the hospital fasting guidelines (6 h for solids, 2 h for clear fluids) and be under the care of the perioperative team in the pre-operative area for minimum of 1 h. Patients were excluded if they were not undergoing elective surgery, were unable to or declined to consent to participate. Potential participants were identified by screening theatre lists. They were assessed for eligibility and approached on the day of surgery. Participants were provided written information and given the opportunity to discuss this with a study investigator. Written informed consent was obtained prior to participation. The full protocol is available from authors on request.

### Interventions

The treatments assigned to the two groups were 15 ml of water (control group) or 15 ml saliva substitute (treatment group) used to rinse the mouth. The saliva substitute was Biotene Dry Mouth Oral rinse (Glycerin, Xylitol, Sorbitol, Propylene Glycol, Acrylic acid, Hydroxyethyl cellulose: GlaxoSmithKline, Australia). The intervention was administered immediately after recruitment and randomisation by an unblinded investigator or member of the pre-operative care team. Baseline measures were assessed prior to participants receiving 15 ml of water or saliva substitute (Biotene) as per group allocation. A volume of 15 ml was chosen as this is the recommended volume for the saliva substitute used. The participants were instructed to rinse their mouth and then spit out any residual liquid without swallowing. The intervention could be repeated after 30 min, though during the trial no participant received a second intervention. Outcomes were assessed 30 min post-intervention.

### Ethics

Ethical approval was obtained from the Northern A New Zealand Health and Disability Ethics Committee (reference number 17/NTA/152). The trial was registered with Australian New Zealand Clinical Trials Registry (ANZCTR number 12618001270202, Date August 27, 2018). Funding for research materials was received from the Auckland Anaesthesia Research Trust who had no role in the design, running, analysis or reporting of the trial.

### Group allocation

Participants were randomised into two groups by block randomisation in groups of eight with an intervention ratio of 1:1 using a computer generated random number. Group allocation was determined by sealed opaque envelopes. Investigators delivering the intervention were not blinded to the group allocation. Blinded outcome assessments were performed by a different member of the research team. Both groups were given their allocation as 15 ml of liquid in a plastic medication pottle. Visually, they looked similar; however, they differed in taste, hence participants were unblinded.

### Statistical analysis and Sample Size

Formal sample size analysis is usually not required for pilot trials [11]. However, a sample size of 60 to 100 per group has been shown in computer simulation to give more reliable estimates of recruitment parameters [12] and statistical parameters required for sample size calculation for subsequent large trial. We have chosen a sample size of 100 as this was deemed sufficient to test feasibility outcomes and provide these estimates. Feasibility outcomes were described with simple descriptive statistics. Scientific outcomes were tested using inferential statistics and an intention to treat analysis. Participant self-rating of dry mouth as reported by VAS was treated as continuous data and tested for normality by visual inspection and application of the Shapiro-Wilk test as our sample size was small. We analysed using a paired *t* test with *p* values and confidence interval reported. A *p* value < 0.05 was taken as statistically significant. These categorical data were summarised using the number and percentage and compared using Mann-Whitney *U* test and chi-squared test for grouped categories. All statistical analyses were performed using the SPSS statistics software (IBM Corp. Released 2017. IBM SPSS Statistics for Windows, Version 25.0. Armonk, NY: IBM Corp).

## Results

**Fig. 1:**
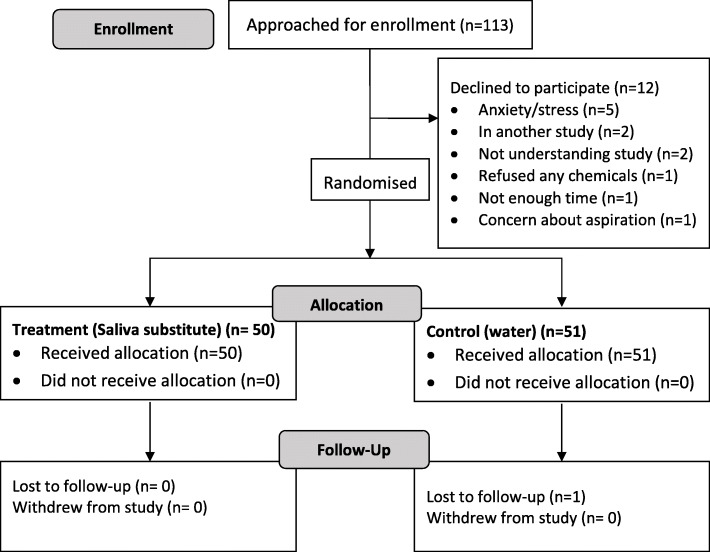
Participant flow

We enrolled at total of 101 participants between October 2017 and February 2018. See Fig. 1 for participant flow.

Patient characteristics are tabulated in Table 1.

**Table 1:** Demographic data

Ethnicity	Control (*N* = 50) *N* (%)	Treatment (*N* = 50) *N* (%)	Total (*N* = 100)
Maori	8 (16)	2 (4)	10
Pacific Island	6 (12)	3 (6)	9
New Zealand European	32 (64)	34 (68)	66
Other European	2 (4)	5 (10)	7
Asian	2 (4)	3 (6)	5
Middle Eastern/Latin American/African	0	3 (6)	3
Sex	Control (*N* = 50) *N* (%)	Treatment (*N* = 50) *N* (%)	Total (*N* = 100)
Male	29 (58)	27 (54)	56
Female	21 (42)	23 (46)	44
Surgical specialty	Control (*N* = 50) *N* (%)	Treatment (*N* = 50) *N* (%)	Total (*N* = 100)
Orthopaedics	16 (32)	24 (48)	40
Urology	14 (28)	7 (14)	21
General Surgery	16 (32)	15 (30)	31
Vascular	3 (6)	3 (6)	6
Neurosurgery	1(2)	1 (2)	2
American Society of Anesthesiologists (ASA) physical status score	Control (*N* = 50) *N* (%)	Treatment (*N* = 50) *N* (%)	Total (*N* = 100)
I	9 (18)	14 (28)	23
II	27 (54)	23 (46)	50
III	13 (26)	12 (24)	25
IV	0 (0)	1 (2)	1
Unknown	1(2)	0 (2)	1
Mean fasting time	Control (*N* = 50) h (SD)	Treatment (*N* = 50) h (SD)	
Food	13.5 (3.1)	13.9 (2.7)	
Fluid	4.7 (3.3)	3.7 (3.2)	

Mean fasting times for food and fluid were 13.7 h (± SD 2.9 h) and 4.2 h (± SD 3.3) respectively. The fasting times between the two groups were comparable.

### Primary outcomes

A total of 113 patients were considered eligible and approached to participate. One hundred and one consented to be part of the study and were randomised. Mean recruitment rate was 6 participants per week. Recruitment of the 101 participants was complete within 3 months. This was in line with our expectation of 5 participants recruited per week given that this was single centre study and the limited availability of recruiters and assessors due to other clinical work load.

The completeness of these data was to be deemed adequate if 95% of all trial endpoints were complete. Of the 101 patients randomised, one did not complete their post intervention questionnaire and was lost to follow-up. Complete sets of data were available on the remainder. Datasets were 99% complete. Only the 100 participants for whom all data was complete were included in the analysis.

We assessed acceptability of the intervention to patients and staff by recording reasons for declining to participate and requesting feedback from the staff involved. Twelve declined to participate for various reasons (see flow diagram). Participant acceptability was inferred from the participant response to the question ‘would you have the treatment again?’ to which > 70% responded ‘yes’. There were no concerns raised by staff regarding acceptability. No side effects or complications were noted. While a higher acceptability rate would have been ideal, being a pragmatic trial a > 70% acceptance rate was above our cutoff of 66% acceptance. Further, no participant withdrew from the study once randomised to receive the intervention.

The study protocol was deemed to have good adherence rates and was easy to follow. The mean time from intervention delivery to post-intervention assessment was used as a marker of adherence. Overall, there was a mean of 30 min (± SD 5 min) between delivery of the intervention and the assessment. Further representation of feasibility targets and outcomes are included in Table 2 below.

**Table 2:** table of feasibility outcomes, targets and actual values

Feasibility outcome	Target value	Actual value
Recruitment rate	> 5 participants a week for duration of trial	Average of 6 participants per week
Participant acceptability of intervention	> 66% of participants would have the intervention again	72% of participants would have the intervention again
Staff acceptability of intervention	No issues raised by staff	No issues raised by staff
Completeness of data	> 95% completeness	> 99% completeness
Adherence to protocol	30mins between intervention and assessment	Average of 30min between intervention and assessment (± SD 5 min)

### Secondary outcomes

The potential effect of the intervention on the participant’s degree of dry mouth was calculated by comparing their pre and post-intervention VAS. ‘Not dry at all’ being 0 mm and ‘worst dry mouth imaginable’ being 100 mm. The difference in millimetres of the position marked on the VAS was calculated for each patient. A positive change indicating an improvement in the degree of dry mouth, and a negative change being a worse dry mouth. These data were analysed using a paired *t* test. The results are presented in Table 3 below. There was no difference in VAS between the groups when an independent *t* test was applied.

**Table 3:** Change in VAS pre and post intervention

Group	Pre-intervention (mm)	Post intervention (mm)	Difference (mm)	Difference CI 95%
Control—mean VAS	33.8	22.6	− 11.2	− 5.1 to − 17.3
Treatment—mean VAS	35.1	22.4	− 12.7	− 6.7 to − 18.7

Participant responses to the question ‘Would you have the treatment again?’ options being ‘no’, ‘yes’ or ‘unsure’ are presented in Table 4. There was no statistically significant difference between the group responses. More participants in the water group than the saliva substitute group indicated they would not have the treatment again (12% vs 6%). The reason for this finding is unclear. However, this most likely represents participants who did not get any benefit from their allocation.

**Table 4:** Participant response to post intervention questions

‘Would you have the treatment again?’	Saliva substitute, *n* (%) *N* = 50	Water, *n* (%) *N* = 50
No	3 (6)	6 (12)
Yes	36 (72)	35 (70)
Unsure	10 (20)	8 (16)
Not recorded	1 (2)	1 (2)
‘How dry is your mouth?’	Saliva substitute, *n* (%)	Water, *n* (%)
Worse	1 (2)	1 (2)
No change	17(34)	22 (44)
Better	22 (44)	24 (48)
Much better	9 (18)	2 (4)
Not recorded	1 (2)	1 (2)
‘How dry is your mouth?’ Combined categories	Saliva substitute, *n* (%)	Water, *n* (%)
Worse/no change/no data	19 (38)	24 (48)
Better/much better	31 (62)	26 (52)

Participant responses to ‘How did your dry mouth feel after the treatment?’ options being ‘worse’, ‘no change’, ‘better’ or ‘much better’ are presented in Table 4. If no data existed, this was to be imputed as worse. The Mann-Whitney test was applied to these categorical data and the chi-squared test to the grouped categorical data. None reached statistical significance. However, when the response categories are combined into ‘worse/no change/no data’ or ‘better/much better’, this represents improvement from 52 to 62%. None of the secondary outcome findings were statistically significant. These data are presented in Table 4.

## Discussion

Dry mouth is a common patient complaint around the time of surgery. There are a number of simple treatments used in other settings which have a positive effect on managing the symptom of dry mouth. Saliva substitutes are a simple, inexpensive and associated with minimal side effects [13]. There are no previous studies at looking at the treatment of perioperative dry mouth and currently, there are no therapies used in this setting for dry mouth. The absolute improvement in ‘better’ or ‘much better’ dry mouth of 10% represents a potential treatment effect that will be tested in a larger trial.

This pilot trial has a number of limitations. The pilot trial was only conducted in a single centre. Although we achieved all of our feasibility outcomes, the success of our protocol may not translate to other centres with different set-ups, procedures and processes. Feasibility measures from the pilot trial will need ongoing assessment in the larger trial. Although information was collected on harm, specifically aspiration, the incidence of this harm is low and may not be apparent in a pilot but may be visible in a larger study. The outcome measures used to test scientific outcomes (VAS and Likert score) have been used to assess dry mouth in oncology patients but not perioperative patients. Group allocation was by opening sealed envelopes. This method may be prone to bias. In the large trial, we will perform site audit and training to make sure researchers are adhering to trial processes. Participants were not told of their group allocation; however, due to the taste difference, their allocation would have been obvious. Knowledge of their allocation may have biased participant’s objective assessment of their dry mouth and treatment effect. The pilot trial was not powered to detect a statistically significant difference for the scientific outcomes.

To our knowledge, this is the first study to investigate a treatment option for perioperative dry mouth. Our pilot study of 101 pre-operative elective surgical patients has indicated that treatment with a saliva substitute is well tolerated with no side effects reported. Although IM DRY was not powered to assess efficacy, we observed a 10% absolute reduction in dry mouth. If this treatment effect were to be replicated in a larger trial, we believe this would be a clinically meaningful difference. We performed an informal poll of 30 colleagues to ask them what they felt was a clinically important effect. The 10% treatment effect was the median response. The 10% relative reduction chosen is arbitrary; however, we feel that 10% (NNT = 10) is a clinically important treatment effect that would be adequate to motivate clinicians to use this inexpensive, simple intervention.

In the IM DRY pilot trial, we demonstrated the feasibility of conducting a larger RCT. Strengths of our study include a high recruitment rate, complete protocol adherence and near complete sets of data. Our protocol was simple to follow and acceptable to investigators, patients and nurses, and would be translatable to a multicentre study.

A larger multicentre RCT (ANZCTR number 12619000132145) is currently underway at four participating sites to assess efficacy of using saliva substitute in the pre-operative setting. The multicentre RCT, BIG DRY, is powered to detect a clinically important improvement proportion (10%) of participants with improved dry mouth. It aims to recruit 838 participants across multiple sites.

## Conclusions

Perioperative dry mouth is an important patient reported outcome (PRO), comparable to pain, anxiety or post-operative nausea and vomiting (PONV). In contrast to these PROs, we have no effective intervention to offer. The IM DRY pilot study successfully achieved its feasibility aims: recruitment rate, completeness of datasets, acceptability and protocol adherence. There were no adverse side effects or complications of the treatment noted. IM DRY suggests there is a positive effect from the use of saliva substitutes to manage dry mouth in pre-operative patients. Saliva substitutes, used in the perioperative management of dry mouth, may be a simple, inexpensive and low risk solution to help alleviate this common complaint. Our findings demonstrate the feasibility of a larger, multicentre RCT to investigate this novel therapy and is currently recruiting in four sites.

## Data Availability

The datasets used and analysed during the current study are available from the corresponding author on reasonable request.

## Abbreviations

ANZCTR: Australian New Zealand Clinical Trials Registry
IM DRY: Interventions to Manage Dry mouth
PONV: Post-operative nausea and vomiting
RCT: Randomised control trial
VAS: Visual analogue scale
